# General Practitioners’ Concerns About Online Patient Feedback: Findings From a Descriptive Exploratory Qualitative Study in England

**DOI:** 10.2196/jmir.4989

**Published:** 2015-12-08

**Authors:** Salma Patel, Rebecca Cain, Kevin Neailey, Lucy Hooberman

**Affiliations:** ^1^ WMG University of Warwick Coventry United Kingdom

**Keywords:** online reviews, physician quality, primary care, Internet, quality, patient empowerment, quality transparency, public reporting, attitude of health personnel, delivery of health care, feedback

## Abstract

**Background:**

The growth in the volume of online patient feedback, including online patient ratings and comments, suggests that patients are embracing the opportunity to review online their experience of receiving health care. Very little is known about health care professionals’ attitudes toward online patient feedback and whether health care professionals are comfortable with the public nature of the feedback.

**Objective:**

The aim of the overall study was to explore and describe general practitioners’ attitudes toward online patient feedback. This paper reports on the findings of one of the aims of the study, which was to explore and understand the concerns that general practitioners (GPs) in England have about online patient feedback. This could then be used to improve online patient feedback platforms and help to increase usage of online patient feedback by GPs and, by extension, their patients.

**Methods:**

A descriptive qualitative approach using face-to-face semistructured interviews was used in this study. A topic guide was developed following a literature review and discussions with key stakeholders. GPs (N=20) were recruited from Cambridgeshire, London, and Northwest England through probability and snowball sampling. Interviews were transcribed verbatim and analyzed in NVivo using the framework method, a form of thematic analysis.

**Results:**

Most participants in this study had concerns about online patient feedback. They questioned the validity of online patient feedback because of data and user biases and lack of representativeness, the usability of online patient feedback due to the feedback being anonymous, the transparency of online patient feedback because of the risk of false allegations and breaching confidentiality, and the resulting impact of all those factors on them, their professional practice, and their relationship with their patients.

**Conclusions:**

The majority of GPs interviewed had reservations and concerns about online patient feedback and questioned its validity and usefulness among other things. Based on the findings from the study, recommendations for online patient feedback website providers in England are given. These include suggestions to make some specific changes to the platform and the need to promote online patient feedback more among both GPs and health care users, which may help to reduce some of the concerns raised by GPs about online patient feedback in this study.

## Introduction

There has been growth in the use of online consumer feedback and review websites (eg, TripAdvisor), which some argue has allowed for transparent information and communication to influence change and has provided opportunities for consumers to read reviews and make more informed choices [1-3]. Similarly, there has been a growth in the volume of online patient ratings and comments, which suggests that patients in England (and other parts of the world) are embracing the opportunity to review their health care online [4-7]. There has also been a growth in the development of online patient feedback, with some patients now reviewing not just their experience of receiving health care, but also their medication and treatment plan [8].

**Figure 1:**
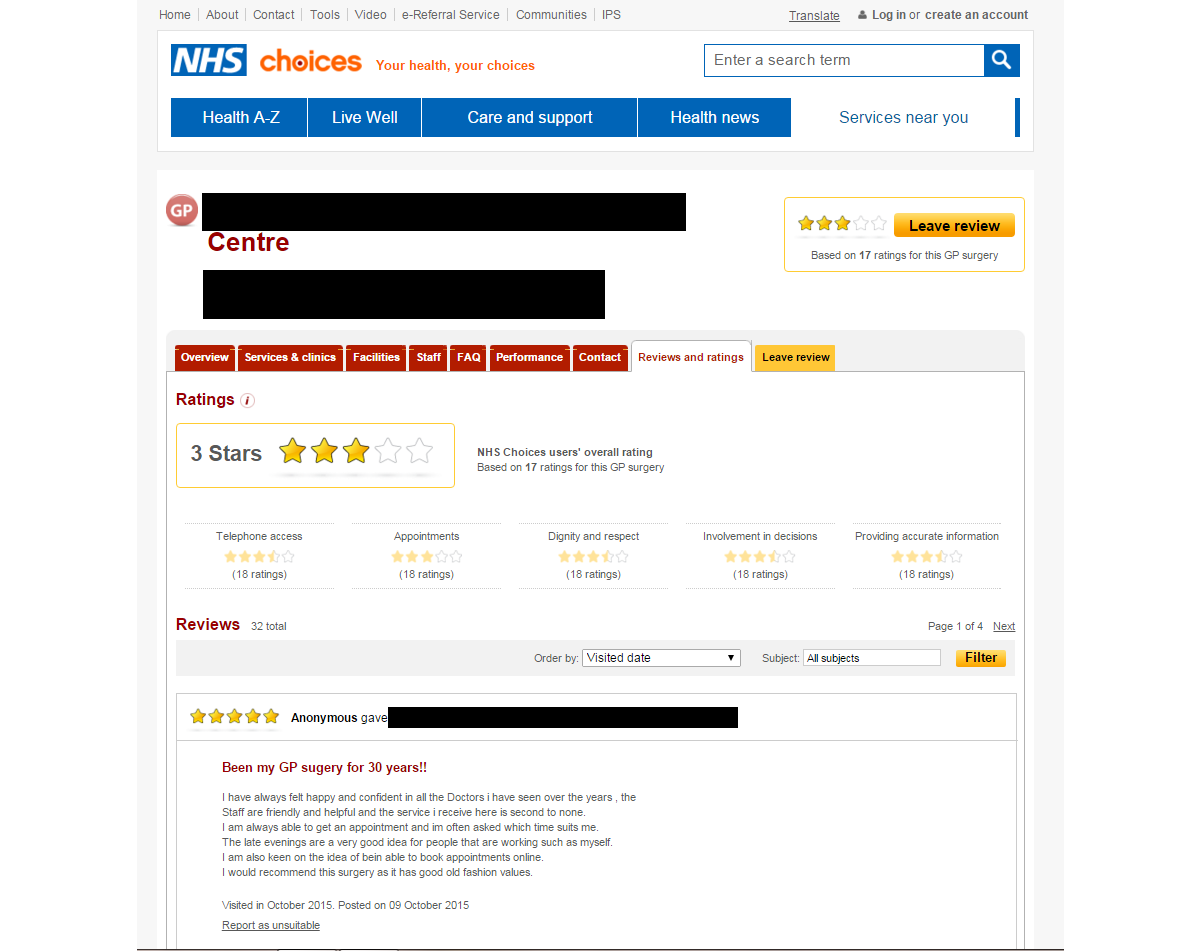
Example of online patient feedback on NHS Choices website.

Online patient feedback, in the context of this paper, can be defined as experiential feedback, ratings, reviews, and comments left by patients, carers, or service users on public Web-based platforms in England, such as NHS Choices, Patient Opinion, and iWantGreatCare, and on apps such as the iPhone-based Great Care app. Users can leave feedback and rate their experience with a general practitioner (GP) service, hospital, dentists, and other health care services, which is available in the public domain for other users to look at (see Figure 1 for an example of an online patient feedback website). The purpose of such websites is to give patients a voice by allowing them to leave feedback online, which some suggest will increase transparency, improve the quality of care, and could be used for service improvement [7,9]. Patients and carers could also then use these ratings and reviews to decide which health care provider to use; in England, this is part of the “patient choice” agenda [10-12].

There are two major websites in the United Kingdom that (1) collect online reviews and ratings from patients about their experience of receiving care from their GP, and (2) allow the public to read patient feedback, which may be used by some patients or carers to choose a GP or a GP practice. The first is the NHS Choices website, which allows patients to leave comments under the GP practice’s name, but does not allow an individual GP to be named or identified in any feedback [13]. The second is the iWantGreatCare website, which allows patients to leave feedback under a GP’s name; therefore, GPs are named and identified in any review or feedback left on this website [14].

Research into online patient feedback or online physician-rating websites has been steadily increasing over the past few years with studies conducted in the United Kingdom [5-7,12,15-19], United States [20,21], Germany [22-26], and Australia [27] adding to the growing literature [28]. There appears to be some evidence to suggest that there is an association between online ratings and quality of care [4,5,21,29,30]. However, the results are not always consistent and, for some studies, the effect size is weak [28]. In particular, the extent to which the online ratings reflect the quality of primary care is less clear [6]. Furthermore, it is difficult to cross-apply the findings of different countries and different online patient feedback platforms because the characteristics of each online platform varies and the culture, context, and policies of each country and health care organization are different [9]. Despite this, some still argue that the “rich source of data” from online patient feedback has the potential to track quality of care [4,5,21,29,30].

Outside of the United Kingdom, a few studies have explored the type of patients who use online patient feedback platforms [24,31-34], whereas other studies have explored the attitudes of patients toward online patient feedback or doctor-rating websites [33,35,36]. A few physician representatives in the media have argued against the introduction of online feedback and rating websites by suggesting that they are dangerous and a waste of resources [37,38], and concerns about slander have also been raised by critics of such platforms [29,38-40]. However, there is very little research that explores health care professionals’ attitudes toward online patient feedback.

Because health care professionals are one of the primary recipients of online patient feedback, one of the aims of this study was to explore and describe GPs’ views about online patient feedback. This paper narrates the concerns raised by GPs in relation to online patient feedback only and the other findings, including the benefits of online patient feedback suggested by GPs, will be reported elsewhere. It is hoped that the findings from this study could be used to improve online patient feedback from the GPs’ perspective and this may help to increase usage of online patient feedback by GPs and, by extension, their patients too.

## Methods

### Data Collection

Because very little is known about GPs’ perceptions of online patient feedback, there was a need for in-depth exploration to capture attitudinal and experiential data; therefore, a qualitative approach using semistructured interviews was best suited. A topic guide was developed following the guidance suggested by Bryman [41], Matthews and Ross [42], and Tracy [43] (see Multimedia Appendix 1 for a copy of the topic guide). A literature review was used as a basis for the topic guide as well as information from discussions with key stakeholders, such as the main lead at one of the online patient feedback website providers in the United Kingdom and 4 GPs. Further issues raised by participants during the interviews were also pursued and participants were encouraged to draw on experiences to illuminate their responses. Three vignettes were also developed (see Multimedia Appendix 2) following the guidance of Barter and Renold [44] and were used as prompts if the participant had not seen an online feedback review website before.

###  Sampling and Recruitment

Participants were recruited from Cambridgeshire, London, and Northwest England. A probability sampling approach was employed initially to ensure a wide range of characteristics of participants as recommended by Bryman [41]. However, despite using various strategies (all described in Table 1), only 6 participants were recruited using probability sampling. Other researchers who attempted to recruit GPs for research in the United Kingdom also reported immense difficulties [45-51]. Therefore, because of the limited response rate and the difficulties with recruiting sufficient GPs for this study using probability sampling, we resorted to using snowball sampling and 14 further GPs were recruited this way using various approaches (detailed in Table 1). In total, 20 GPs were interviewed for this study because at that point thematic saturation [52] had been reached.

**Table 1:** Recruitment strategies and number of general practitioners recruited for this study.

Recruitment strategy		Number of GPs recruited
**Probability sampling**		
	Direct invitation to GP (postal invitations were sent to 25 practices in Cambridgeshire, which were then followed up by phone calls. A primary care research network [PCRN] also sent a letter on our behalf inviting and promoting the study to research active GPs and practices in Coventry)	1
	Invitation through practice managers (phone calls were made to 25 GP practice managers in Cambridge and a follow-up email was also sent; 13 further GP practices were then contacted through phone and then fax)	2
	Promoting study in email-based GP newsletters (the study was promoted in the following GP newsletters: Cambridgeshire NHS Newsletter, Lewisham Weekly Newsletter, Lambeth PCT newsletter, South NHS North West London Newsletter, Participate Autumn Magazine)	3
**Snowball sampling**		
	Email to acquaintances with potential GP contacts	1
	Twitter call out to acquaintances with potential GP contacts	0
	GPs emailing their GP acquaintances	5
	Medical doctors phone calling their GP acquaintances	8
Total		20

###  Study Interviews and Participants

The interviews were conducted at the location that was most convenient to the participant, with a preference given to the GP practice where the GP worked. However, some GPs preferred to be interviewed at their home outside of working hours and one GP was interviewed at a private meeting room. GPs were paid £80 for their participation.

All participants were sent the participant information sheet beforehand either through email or in the post and this contained the aims of the study among other things. Informed consent was obtained from all participants before the start of the interview. Interviews were recorded using two digital voice recorders. The study had full ethical approval from the Biomedical and Scientific Research Ethics Committee at the University of Warwick.

The descriptive characteristics of the 20 participants interviewed are shown in Table 2. Although 60% (12/20) of the GPs interviewed were between the ages of 30 and 34 years, they represented a variation in relation to duration of experience as GP, type of GP, and gender.

**Table 2:** Demographics and practice characteristics of participants (N=20).

Baseline characteristic		Frequency, n (%)
**Age (years)**		
	25-29	1 (5)
	30-34	12 (60)
	35-39	3 (15)
	40-44	1 (5)
	45-49	1 (5)
	50-54	1 (5)
	55-59	1 (5)
**Gender**		
	Male	12 (60)
	Female	8 (40)
**Type of GP**		
	Salaried	6 (30)
	Partner	7 (35)
	Senior partner	2 (10)
	Lead	1 (5)
	Locum	4 (20)
**Years practicing as GP**		
	1-5	14 (70)
	6-10	2 (10)
	11-15	1 (5)
	16-19	1 (5)
	≥20	2 (10)

###  Data Preparation and Analysis

Interviews were transcribed verbatim and each transcript was double-checked for inaccuracies. The transcripts were then exported to NVivo software and analyzed using the framework method. This is a form of thematic analysis developed in the 1980s by researchers at the NatCen Social Research and has been used widely since then, both in policy research and other areas [53-56]. A thematic framework was created which was refined and then applied to all the data. Categories and themes were refined and defined until explanations were formed and thematic maps were produced. The analysis was conducted by the first author (SP) and the thematic framework and thematic maps were checked by all authors.

## Results

In this interview-based study, participants were asked about their experience, usage, and attitudes (both positive and negative) toward online patient feedback. Because of the richness, depth, and breadth of the interview data, it was only possible to report the concerns about online patient feedback raised by participants in this paper. Other findings, such as those related to GPs’ perceived benefits of online patient feedback or their attitudes toward social media, will be reported elsewhere. However, to place the GPs concerns into context, we believe it is important to understand participants’ usage of and experience with online patient feedback as well as their overall impression of patients leaving feedback online about them. This is narrated in the subsequent section.

###  Usage, Awareness, and Overall Impression of Online Patient Feedback

Three-quarters of GPs interviewed in this study were aware that patients can leave feedback for them or their practice on the NHS Choices website. Four GPs had direct experience with online patient feedback and their practice or GPs in their practice had received feedback online on the NHS Choices website. One of the GPs also admitted he had received negative personal feedback online on the iWantGreatCare website. The majority of GPs interviewed (n=17) did not currently consider online patient feedback as a way of collecting feedback from patients. However, 12 participants believed that patients do have a right to place feedback about their GP online as long as the feedback was factually correct and on an appropriate website. Five participants, however, disagreed suggesting that patients do not have the right to place feedback about their GPs online.

### Concerns About Online Patient Feedback

**Figure 2:**
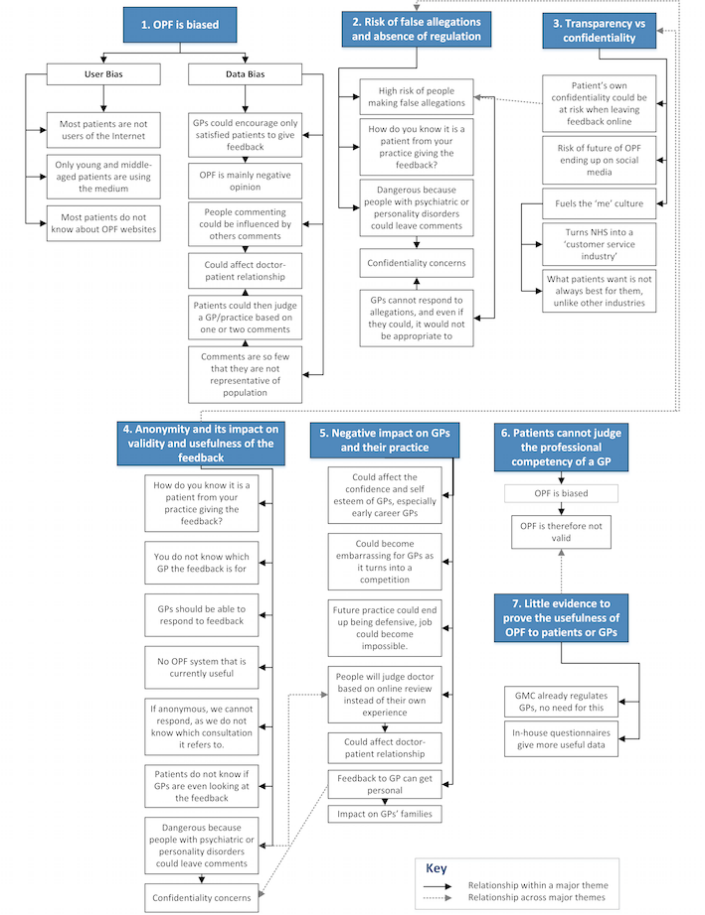
A thematic map illustrating the downsides of online patient feedback (OPF) according to the GP participants in this study.

In this study, 56 individual concerns were raised by GPs (31 of which were unique) when asked the open-ended question: “Do you have any concerns about online patient feedback?” Other concerns raised about online patient feedback during the interview were also included in the analysis. Figure 2 is a thematic map that shows a summary of the concerns raised by participants and the 7 main themes (highlighted and numbered in the diagram) are discussed in the subsequent sections.

### Theme 1: Online Patient Feedback is Biased

#### User Bias

Most participants were concerned that it is only young and middle-aged patients who are leaving feedback on online patient feedback websites. Some participants commented that the majority of their patients were elderly and were certainly not using this medium. This, according to them, indicates that the feedback and ratings that are online are not representative of the overall experience of their patients and, therefore, they questioned its validity and usefulness:

You are getting your opinions from again, one particular sector of the community...their perceptions, their understanding and attitudes are different to the rest of the population...You have to use it with a bit of scrutiny there, in terms of interpreting the data, how do you put that in practice? If you were to put in any changes?

P10

One participant felt that there was not enough publicity about the NHS feedback website and many patients do not even know about the website.

#### Online Patient Feedback is Mainly Negative Opinion

One of the most repeated concerns raised by a quarter of participants was that online patient feedback is mainly negative opinion and is and will become a channel for disgruntled patients:

There’s a bias towards putting negative feedback, if they’ve had a good experience, nonoutstanding one but an adequate one, they have no complaints but their needs are made [sic], they are unlikely to go on and post positive feedback.

P13

Other participants argued that it only takes one negative feedback to harm a GP’s reputation:

One unfortunate comment or bit of a negative feedback, which may be taken well out of context, can harm your reputation.

P16

#### Online Patient Feedback is Too Small in Number

A few participants (n=3) raised concerns that feedback left for a GP or a GP practice on online websites is too small in number and, therefore, it is not representative of their record of performance:

It’s a small number of comments, we’ve had 2 [online reviews] out of a population of 12,000 [on the NHS Choices website], I don’t think that would be representative enough for a potential patient to go on and go, “alright ok they’ve got 50% bad comments, right I’m not registering there” (laugh).

P2

GPs were concerned that patients could judge a practice or GP based on a very small number of reviews (and make an invalid “choice”) and this could also affect the doctor-patient relationship.

#### Reviews Could Be “Gamed”

Some participants (n=5) were concerned that reviews could be manipulated and that some GPs could encourage only satisfied patients to give feedback, which again would add bias to the data. Moreover, 4 participants from this study admitted they would only encourage those patients who they know will give positive feedback to leave feedback online:

No, obviously, if somebody has had a good experience, you might encourage it [leave feedback on NHS Choices]. But also, I think if somebody wants to make a complaint I would say you can write to the practice manager and there’s always, I may not actively promote it [giving feedback on NHS Choices].

P7

### Theme 2: Risk of False Allegations and Absence of Regulation

Half of the participants felt that there was a very high risk of patients leaving false allegations about them or the practice on online patient feedback websites. Furthermore, a quarter of participants felt that the owners of such websites (eg, the NHS Choices feedback site) were not regulating feedback left on these websites and removing malicious or factually incorrect comments from patients. Participants were particularly concerned that their patients with psychiatric or personality disorders could leave factually incorrect or malicious comments about them and harm their reputation:

You will have everyone, including people with severe psychiatric illness [leaving feedback on online patient feedback websites]...so I think it’s [online patient feedback] potentially quite [a] dangerous tool.

P19

Also, a few participants (n=3) felt that even if GPs could respond online to such allegations, it would not be appropriate for them to respond online.

### Theme 3: Transparency Versus Confidentiality

Eight participants agreed that patient reviews left online will seemingly help to increase transparency of care and improve the quality of care, and they were not concerned about the feedback being online and being so transparent as long as there was a “proper system” in place for online patient feedback:

It worries me if it’s [online patient feedback] not a proper system.

P18

By “proper system,” participants meant that the website was well regulated and validated. The website could verify, for example, that the patient leaving the feedback was an actual patient of that particular GP or that the patient did not have a malicious agenda. However, 8 participants were concerned about the platform being “too open” (P11) and in public due to the possibility of people making false allegations, and its damaging impact on the reputation and career of a GP and a GP’s own personal confidentiality:

If it’s [feedback] in public, particularly if I felt it was untrue...if you got y’know someone made an allegation...if that happened to a doctor it could destroy their career, and their self-esteem, and I just think that’s not fair on doctors.

P1

A few participants felt that these types of websites would fuel what they called the “me” culture and turn the NHS into a “customer service industry” and were concerned that it could lead to patients in the future thinking it was perfectly fine to leave feedback about physicians on social media, where according to them, it is impossible to validate or regulate the feedback. Another participant went on to explain that, particularly in health care, what a patient wants is not always what is best for them:

There can be a difference certainly between what people need and what people want, and if people don’t get what they want, often they can feedback negatively about that, even though actually the doctor or the medical provider or whatever who is looking after them, has done exactly the right thing.

P2

In addition to worrying about the confidentiality of GPs themselves, a few participants were also concerned that the transparent nature of the feedback meant that a patient’s own confidentiality may be at risk because they may feel the need to disclose personal health information about themselves on a public platform. Some participants (n=3) were also concerned that GPs are unable to respond to patient reviews online due to the possibility of violating doctor-patient confidentiality because they may need to disclose health information about the patient in their response.

### Theme 4: Anonymity and its Impact on Validity and Usefulness of the Feedback

All participants (N=20) were aware that the feedback left on NHS Choices was left anonymously by patients. Some participants raised concerns that because the feedback was left anonymously, they would not know which consultation it referred to and, therefore, could not respond to the feedback nor make real use of it for improvement. Others (n=5) felt that the anonymous nature of the feedback meant they would not know if it was an actual patient from the practice that had left the feedback and questioned whether such feedback is even valid:

Again, if it is anonymous, then, with any feedback really, you really don’t know, is it somebody from this practice, or somebody, well it could be anybody really leaving a feedback there [on online patient feedback websites].

P10

Participants were then asked specifically if the feedback would be more useful to them if it was not left anonymously. Seven participants said that it would be more useful to them if feedback was left with the patient's real name so that they can then look up the consultation and see what went wrong:

If you had their name there, you could obviously understand where this is coming from, and then you can think about it or go back on it, and make ways of improving yourself during your consultation skills. But if it is very anonymous...out of seeing 40 patients in a day, 200 in a week, which one are we talking about, in terms of who?

P10

However, the remaining 13 participants disagreed, commenting that it would not be fair on patients to give their real name because, according to them, it will affect the doctor-patient relationship and patients will not leave feedback online if they cannot leave it anonymously. One participant appeared to suggest a solution that patients should leave their NHS number when they leave feedback to verify that they are a patient registered at that particular practice. Another participant raised the question that despite patients not naming themselves when leaving feedback online, would patients really remain anonymous because sometimes it was easy to identify a particular patient from an anonymous online comment.

### Theme 5: Negative Impact on General Practitioners and Their Practice

In addition to the threat of defamation discussed previously and its impact on GP reputation and career, 2 participants were also concerned that negative feedback online could affect the self-confidence and self-esteem of GPs, which would in turn affect their practice, especially those GPs who are early in their career:

It [online patient feedback] will affect people in their early career a lot more, and could break their confidence and make them insular. Is that what you really want to be doing to your future doctors?

P11

Some participants also felt that people will start judging GPs based on online reviews instead of their own experience and this could also affect not only the doctor-patient relationship, but also their practice. Furthermore, participants raised concerns that due to the possibility of negative reviews going online (whether true or false), future practice could end up being defensive and it would be impossible to practice properly:

I don’t know how on earth we are going to have a decent relationship...doctors have become so defensive already...just to make sure they don’t get things online, or do you want them to actually do right for you...give you good care in the right manner in the right timeframe, in a manner which is satisfactory to, or do want them to just do things because they are so scared of litigation of online feedback.

P11

Other GPs raised concerns that it could become embarrassing for them if their practice became public and turned into a “competition” and this could impact patient care too. One participant was particularly concerned about the negative impact online patient feedback could have on her family:

I suppose it’s just the fact that something that’s online...you think about your family and other people, close to you nearest and dearest, sort of looking at things and getting upset on your behalf as well.

P12

### Theme 6: Patients Cannot Judge the Professional Competence of a General Practitioner

Some of the participants who were not in favor of online patient feedback argued that the General Medical Council was already regulating them, so there was no need for patients attempting to “regulate” them online and, in fact, how can patients judge whether a GP was competent or not?

Can you really say a patient has that ability to say whether you are underperforming or not?...so the people that are doing appraisal and revalidation are also GPs, they know what you should be doing. I think they should police it, as opposed to patients.

P14

### Theme 7: Little Evidence to Prove Usefulness of Online Patient Feedback to Patients or General Practitioners

Two participants argued that there was no evidence currently to prove the usefulness of online patient feedback to patients or GPs:

I think some things with Government policy or in the NHS policy are brought in without having any evidence of benefit, sometimes people jump at the chance “oh we will do this” and they don’t think why.

P1

Furthermore, a few participants argued that existing methods of collecting patient feedback, such as in-house questionnaires, were perfectly adequate and gave more useful data. However, when asked separately about offline feedback, more than half of the participants commented that they did not collect “useful” data.

## Discussion

To our knowledge, this study is the first study that explores GPs’ concerns about online patient feedback. The study’s findings suggest that GPs have reservations about online patient feedback and question online patient feedback’s validity, value, usability, and its transparent nature, and are worried about how this will impact them, their practice, and their patients.

### Validity and Value of Online Patient Feedback

In this study, GPs were concerned—among other things—about the bias of online patient feedback, both from the user and data perspective. The concern of potential bias due to the age of patients using online patient feedback (in favor of younger patients) has also been raised in literature by some [39,57-59] and some studies appear to support it [6,31,33,36]. However, it could be argued that even patient satisfaction results that are offline are influenced by age, education, and health status [60]. Furthermore, Greaves et al [5] argue that although there may be risks in using ratings from a small group of self-selecting patients, according to them it is outweighed by its positives, mainly that online patient feedback is low cost and has the ability to detect episodes of poor care that a traditional survey may miss. However, as some participants highlighted in this study, this does not address the question of whether a rating of a particular GP or a GP practice can truly be representative, valid, and fair if only the younger and middle-aged patients are leaving ratings or feedback. This is especially crucial for those practices and GPs that serve a largely elderly population.

Furthermore, other participants argued that online patient feedback is mainly negative opinion and will become a channel for disgruntled patients. This sentiment has been raised in opinion articles [37,39] and literature [29,61], but has been counteracted by the argument that many studies (including [6,19,20,22,29,34,62-66]) have found that the majority of feedback left on online physician review websites is actually positive [28]. However, Greaves et al [6] found that the recommendation level of GP and practices in England for the same period was 64% online and 82% in patient surveys. This, they suggest, does indicate that there may be a selection bias in online patient feedback toward less satisfied patients versus when patients are selected randomly and this appears to suggest that the concerns raised by participants in this study may be valid. Furthermore, Merrell et al [61] argue that the abundance of positive reviews cannot negate the impact of negative ones because negative ones, however few, can have long-lasting ramifications as a few GPs in this study highlighted. This is also supported by findings from a study by Hanauer et al [67] who found that parents who are exposed to a positive recommendation of a physician from a neighbor are less likely to choose that physician for their child if they were then exposed to negative reviews about that same physician online. However, Adams [68] found that patient reviews online are not always inherently positive or negative; rather, they contain a mixture of positive and negative comments as well as references to and comparisons with previous health care experiences.

Another argument put forward by GPs in this study was that patients cannot judge the professional competence of a GP; therefore, how can online patient feedback be a true representation of their practice? The concern of whether a patient can adequately judge quality of care received was also raised by Lagu and Lindenauer [58]. It would be useful to explore in future research whether patients are aware that patient-led ratings may be based primarily on the bedside manner of a GP, according to some GPs in this study, and not necessarily on the clinical competence of a GP.

The GPs interviewed were also concerned that feedback left online for them are too few in number and therefore not representative of their performance. This appears to be supported by a study on the NHS Choices feedback website, which found that only 61% of GP practices in England had been reviewed and the number of ratings left per practice was variable with an average of only 2 ratings per practice [6]. However, this study by Greaves et al [6] explored data from more than 5 years ago (between October 2009 and December 2010) and more up-to-date analysis of such websites is required to truly understand the current state because usage may have changed. Taking into account that reviews on the NHS website only correspond to 0.005% of all GP consultations [6] and that studies from the United States [29,66,69,70], Germany [34,71,72], and Australia [27] all indicate that less than 30% of physicians have been rated (and even those that have been rated have on average less than 4 ratings each), the assertions raised by the participants in this study may be true and valid, and need to be addressed by online patient feedback platform providers.

Strech [40] suggests that the solution to this may be that ratings should not be made available until they reach a certain baseline number (eg, 5-10). Although individual pieces of feedback could be displayed before a baseline number is reached, the overall star rating, for example, should not be shown until there are a reasonable number of ratings left for a practice or GP. If the NHS and other online patient feedback website providers want GPs to take these reviews seriously and for the ratings per practice to be “valid” and representative (so that patients can make an accurate “choice”), it needs to do more to get patients to leave reviews (see Textbox 1 for a list of recommendations for online patient feedback providers based on this study). Although the overall rating may not be representative of the quality of care provided by GPs at a GP practice, this does not mean that the individual patient feedback left, however few, may not be useful to GPs and practices to use to make changes and identify opportunities for improvement. This suggests that even if some feedback providers may choose not to publish reviews until a certain baseline has been reached, the unpublished reviews could be sent to GPs to review and use for improvement.

***Recommendations for online patient feedback website providers based on findings from this study*.**

Based on the findings from this study with GPs, recommendations for online patient feedback website providers in England are as follows:

1. Promote online patient feedback more among GPs and patients

- Promote online patient feedback among GPs and train GPs to use online patient feedback. This will help to reduce misunderstandings about online patient feedback among GPs, which may help to increase usage of online patient feedback by GPs and also by patients. If GPs believe online patient feedback is valid and useful, they are more likely to promote it to their patients and this may be one of the most effective ways to promote the platform with patients. This could be done through training or may even be as simple as creating a document entitled “A Guide to Online Patient Feedback [specific platform name] for GPs” and signpost it well, both online and offline.
- Implement a campaign to promote online patient feedback to patients. This will help to increase the number of patients and type of patients leaving feedback and reviews and therefore the feedback left online is less likely to be biased and unrepresentative. This may mean GPs will take it more seriously and patients will be able to make a more valid “choice.” This could be done through traditional marketing routes through GP practices and digital methods, such as social media and TV ads.

2. Convince and reassure GPs about the value of online patient feedback

- Outline precisely how feedback left on the website is moderated and regulated, especially in relation to malicious or personal comments about individual GPs.
- Outline on the website and in any marketing leaflets what GPs can do with feedback that is left online for them, in particular, how to respond to it and use it for improvement.
- Make patients aware that feedback and ratings left by other patients on these online patient feedback websites may be based primarily on bedside manner and that the majority of patients do not have the ability to judge the professional competence of a GP.

3. Consider some changes to the online patient feedback website

- To eliminate concerns about patients judging a GP or a practice based on just a handful of reviews, have a larger number of reviews on the website per practice before the overall rating is calculated and shown.
- Validate that the patient leaving feedback on the online patient feedback website is registered as a patient at the given practice through, for example, asking the patient for his/her NHS number. The NHS number could be concealed from the practice to protect the identity of the patient.
- Allow patients to leave feedback both for individual GPs and for the practice.
- Create an aggregated score of results of measures of competence of GP and patient feedback and reviews, left online and offline, instead of the rating being based on just a few reviews left online at the moment. This has been recommended by the Nuffield Trust [73].

The limited number of online reviews for GPs and practices may be partly explained by one participant’s comment that “patients do not even know about online patient feedback.” This appears to be supported by a study that found that only 15% of the 200 participants in one borough of London were aware of the existence of online feedback websites in health care [36]. However, this study was conducted almost 3 years ago and the awareness of online patient feedback among Londoners may have increased. Nevertheless, there is little evidence as to what extent the NHS Choices feedback website is known and used by patients in the United Kingdom. However, in the United States and Germany, recent studies found that approximately a quarter of respondents had used a physician-rating website [24,33]. This may be partly due to the higher usage and popularity of private health care in both the United States and Germany.

Even when reviews or feedback are left for GPs, some GPs in this study were concerned that the feedback or reviews left by patients could be manipulated without the GP or the practice doing anything “illegal” and this could add serious bias to the data and it would question the validity of the overall rating. This concern is similar to concerns raised in literature that ratings could be “gamed” by organizations or individuals and people could leave fake or multiple entries [6,58,74]. Lagu et al [66] analyzed feedback on review websites in the United States and found several reviews they felt had been written by the physician because they contained information only the physician would know. In another study, Kadry et al [63] found some reviews that they believed were acts of sabotage from competing providers.

A few other GPs in this study argued that there was not enough evidence to prove the usefulness of online patient feedback to patients or GPs. Although research into online physician-rating websites has been steadily increasing over the past few years and studies conducted in the United Kingdom, United States, Germany, the Netherlands, and Australia are all adding to the growing literature [4,5,21,29,30], there is currently a huge gap in the literature. For example, further research is needed to determine whether patients believe online patient feedback is “useful” to them to give feedback or to use to choose a health care provider.

### Transparency of Online Patient Feedback

The transparent nature of online feedback websites is what has made it so attractive to patients and health policy makers because the understanding is that reviews left online will apparently increase transparency of care and improve the quality of care [63,68]. A few participants in this study appeared to support this view, whereas the majority had concerns about the platform being “too open” (P11) and in public due to the high risk of people making false allegations and its damaging impact on the reputation and career of a GP, a GP’s self-confidence, self-esteem, and personal confidentiality, all of which could affect their professional practice. Concerns about slander have also been raised by critics of online patient feedback platforms, mainly physician representatives such as the British Medical Association [29,38-40]. However, NHS Choices in England claim to have a strict set of regulations that they use to protect physicians and hospitals from content that may damage their reputation [13]. Despite the NHS Choices promising that all “inflammatory remarks” are removed, it is unknown how this is put into practice and to what extent, and also what constitutes “inflammatory.” Owners of such websites need to make this clear to their users [40]. Furthermore, a few participants in this study remarked that although NHS Choices may anonymize the doctor, it was easy for GPs and the public to work out which GP or staff member the comment was directed to; therefore, it does not really give them the anonymity and protection it claims to.

### Usability of Online Patient Feedback

Others remarked that due to the GP being anonymous in comments, it was difficult to work out who the comment was for and, therefore, could not be used for improvement. This concern was also raised by McCartney [39] who as a practicing GP felt that it was difficult, if not impossible, for doctors to learn from anonymous comments. One participant explained that in his opinion the difference is related to the size of the practice; where there are fewer GPs in a practice, it is easy to work out who the feedback is for and their reputation could be harmed much more easily. Another participant felt that harming of reputation was happening offline too, so it made no difference whether it was online or offline. However, others remarked that being online was “too public” and hundreds and thousands of people could have access to it. Some physicians have gone as far as getting a court order to remove an online review according to Kadry et al [63], but they argue that it is very difficult to defend against online misinformation and defamation. Further research is required to determine how patients feel about remaining anonymous and naming their GP online when leaving feedback about their GP online and whether remaining anonymous and naming their GP are key criteria for them to leave feedback on online patient feedback websites.

### Conclusions and Recommendations

The majority of GPs interviewed in this study had concerns and reservations about online patient feedback because they felt that online patient feedback was not an accurate representation of their performance due to user bias and data bias. They were also worried about the impact this could have on them, on their practice, and their patients, who may use these “questionable online ratings” to make an “invalid choice” of which health care provider to use. GPs in this study also felt that due to the transparent nature of the feedback online and what they perceive to be lack of regulation, there is a high risk of false allegations being left about them, which could have an impact on them personally, on their family, on their professional practice (more defensive medicine), and on their relationship with their patients. Other GPs questioned the usefulness of the online feedback if the feedback is left anonymously, but acknowledged the benefits to patients of leaving feedback anonymously. A few participants also argued that there was no current evidence to prove online patient feedback’s usefulness to GPs or patients.

Our findings suggest that most concerns raised by GPs may be valid and need to be addressed by online patient feedback providers and other online patient feedback stakeholders. If the NHS and other online patient feedback website providers want GPs to take these reviews seriously, for example, and for the ratings per practice to be valid and representative (so that patients can make an accurate “choice”), they need to do more to get patients to leave reviews. Promoting online patient feedback among GPs and reassuring them of the safety and usefulness of such platforms may also mean GPs are more likely to use online patient feedback for their own professional development and encourage their patients to leave feedback on online patient feedback websites. Other recommendations for online patient feedback website providers based on findings from this study can be found in Textbox 1.

### Limitations of the Study

One of the aims of this descriptive study was to explore GPs’ concerns about online patient feedback and the qualitative findings from this study were not intended to be representative of all GPs in England. We do acknowledge that the sample size for this study was small (N=20) and because 60% of participants were between the ages of 30 and 34 years, there may have been a sample bias toward more technology-savvy GPs. However, we found little difference in Internet usage of all the different-aged participants in our sample. We did attempt to recruit participants randomly to get GPs from different age groups and backgrounds, and more than a quarter of our participants were recruited using probability sampling.

Despite our findings not being representative of all GPs in England and this paper being limited to narrating GPs’ negative attitudes to online patient feedback only, the findings highlight key concerns related to online patient feedback from GPs’ perspective and place them into the context of existing literature and viewpoints. This helped form recommendations for feedback providers and can help inform further research in this area.

## Appendix

Multimedia Appendix 1 A copy of the complete topic guide used for the interviews in this study.

Multimedia Appendix 2 A copy of the vignettes used during interviews with the GPs.

## Abbreviations

GP: general practitioner
