# Supplementary material for: General Practitioners’ Concerns About Online Patient Feedback: Findings From a Descriptive Exploratory Qualitative Study in England
Source: J Med Internet Res. 2015 Dec 8;17(12):e276. doi: 10.2196/jmir.4989 (PMC4704896; doi:10.2196/jmir.4989)
Supplement: Multimedia Appendix 1 [file jmir_v17i12e276_app1.pdf]

Multimedia Appendix 1

Topic Guide

Interview: GPs' attitudes towards online patient feedback

1. Introduction

Introduction to research: nature and purpose, confidentiality and permission (consent form signed) – Put recorder on now.

2. Generic Participant background

Name/sex/age/professional role of participant/years of practicing

3. Internet Use

Use and frequency of: Internet/social media/rating websites such as TripAdvisor & amazon/for decision making and rating/smartphone/apps

4. Generic patient feedback tools: experience & attitudes towards them

- **Current methods** used in the surgery to collect patient feedback – do they collect valuable data? If they don't collect useful data, why don't they? Which tools give data that can lead to improvement of their own practice?
- **Significance** of patient feedback – Do you think it is important to collect patient feedback? How important is it to use patient feedback? What could it be used for?
- **Encouraging patients to give feedback –** Do you think the NHS should encourage patients to give feedback on the care they've received? Do you ever encourage patients to give you feedback?
- **Receiving positive/negative feedback –** how does that make you feel?
- **Response to generic patient feedback –** Have you ever responded to patient feedback? If so, how and why?

- **Use of generic patient feedback** – Have you ever used the data? If so how?
    - Can the data be used for professional development? How and why?
    - Have you informed patients of your use of their feedback? How and why?
- **Real-time feedback** - Real-time feedback is when patients give feedback as soon as they've seen you. Would you encourage your practice to implement a real-time feedback tool?

5. Online feedback: experience & attitudes towards them

- **Experience of online feedback:** Do you know what online patient feedback is? *(If not, researcher will explain briefly)*
    - **As a patient:** Have you ever given/used online patient feedback? Would you consider using online patient feedback to make a choice of service provider or clinician? Do you think it's a useful way to decide which service provider or clinician to choose?
    - **As a professional:** Are you aware if you've received online patient feedback? If so, what did you do with it? Did you use it, and how? If ignored, why?

- **Impact of online patient feedback on GP**
    - Would you prefer to receive a formal complaint about you offline rather than online feedback? Why?
    - Do you think there's a difference between negative feedback and complaints?
    - Does the practice have a different procedure for complaints & feedback?

- How does receiving positive patient feedback in public make you feel? (May use vignette 2 to demonstrate what positive feedback is)
- How does receiving negative patient feedback in public make you feel? (May use Vignette 1 to demonstrate what negative feedback is)
- Do you think patients have a right to give you feedback on your performance as a GP? Do you think they have a right to place it online?
- Some patients may argue that they have a right to know if providers are sub-standard. How do you feel about that?
- Some patients may say that doctors should be positive about receiving feedback to identify opportunities for improvement. How do you feel about that?

- **Perceived concerns: Do you have any concerns about online patient feedback?**

  Possible prompts:
  - Does the transparent and public nature of the feedback targeted towards you or the practice concern you or worry you? Why?
  - Do you think your patients would rate you not only based on your professional skills but also as a person? (Does this worry you? Why?)
  - Do you think a negative review read by a patient of yours may prevent a previously good doctor-patient relationship from continuing with the same ease? Why?

- **Perceived benefits: Can you see any benefits in relation to online patient feedback vs other feedback methods?**

Possible prompts:

- Patients argue that that they give feedback because they have a desire to assist in improving the service. Do you think this is true? Why?
- Do you think anonymous feedback that is online may provide new insights on the perceptions and needs of your patients? Why?
- Some argue online patient feedback will force doctors to improve their performance and bedside manner out of fear that patients may post online about them. Do you think it will ‘force’ GPs to improve performance? Why?
- Do you think your patients would have more confidence in you if they read positive reviews of yours online? Why?
- Some argue that openness leads to increased trust between doctors and those they care for, and a relentless increase in quality health outcomes. What do you think about that?
- Do you think patients are less likely to send an official complaint about you if they are encouraged to give anonymous feedback online?
- Do you think your patients would find it easier to give anonymous feedback online than paper based anonymous feedback?

6. Using online feedback

Response

- Should a GP respond to critical feedback? (may use vignette 1 to demonstrate)
- Should a GP respond to positive feedback? (may use vignette 2 to demonstrate)

- In which situations would you consider responding to online feedback?
- Do patients have a right to receive a response to the feedback they leave? Why?

Use

- Have you ever used online feedback? How?
- Would you use online feedback to change your practice? If not, why not? If yes, how and why?
- Currently reviews are anonymous on NHS Choices. Would there be more useful to you if they weren't? Why?

7. Feedback to practice vs. feedback to GP

- Currently there are two major websites in the UK where patients can give feedback to the GP. One website is where they give feedback and it is left under the practice name, and the second is where they leave feedback under a particular named GP. Which do you prefer/feel more comfortable with?
- Some argue that to support continuous self-improvement requires individual data, not an average score or feedback for the whole department or practice. Would you agree? Why? Which one would you feel more comfortable with? Why?
- (Possible question: A GP may be getting a low score due to something not related to him, for example hypothetically the patient may be annoyed about the time allocation. *(Possibly show Vignette 3 as this point).* Would this have an effect on whether you would prefer to receive personal feedback instead of practice based feedback?)

8. Future of online feedback

- What are your thoughts on the idea that the collective voice of healthcare users, not the powers of the traditional healthcare regulators, becomes the new health watchdogs?
- **Online feedback stories vs. surveys** – Do you think stories that narrate the patient experience are more likely to lead to changes in your behaviour or practice than say the results of a survey?
- Would you ever encourage your patients to give online feedback? Why?
- Do you think if you or your surgery encouraged patients to give online feedback you would be looked upon more favourably? Why?
- Do you think this service (the NHS Choices service) should remain or should be scrapped, or should change in any way? If so why?
- Do you think the iwantgreatcare website should remain or should be scrapped, or should change in any way? If so why?
- Where do you see the future of online patient feedback?

9. Social Media and Feedback

- Does your practice have a social media presence? If so, what does it use it for?
- How do you feel about feedback left on Facebook page or on twitter? Any plans to respond to it?
- Would you ever ask for feedback using social media? Why?
- Do you think if you or your surgery engaged with patients using social media you would be looked upon more favourably? Would it benefit you/patients? How? Why?

- Do you think if you or your surgery encouraged patients to give feedback using social media you would be looked upon more favourably? Would it benefit you/patients?

- Do you think there is a difference between leaving feedback on NHS Choices for example and leaving feedback on social media?

I've had difficulty recruiting GPs to take part in this study, and I'm really grateful you agreed to take part. May I ask what motivated you to take part in this research?

10. Ending –

- Any further things you'd like to add
- Thank you
- Inform them of payment
- Leave contact card.
