# Supplementary material for: General Practitioners’ Concerns About Online Patient Feedback: Findings From a Descriptive Exploratory Qualitative Study in England
Source: J Med Internet Res. 2015 Dec 8;17(12):e276. doi: 10.2196/jmir.4989 (PMC4704896; doi:10.2196/jmir.4989)
Supplement: Multimedia Appendix 2 [file jmir_v17i12e276_app2.pdf]

Multimedia Appendix 2

Caption: A copy of the vignettes used during interviews with the GPs

Vignette 1

Study A

An example[1] of feedback left on NHS Choices

What else can I say..

Eve visited [redacted] in April 2012.

Overall rating

I would not recommend to a friend

I am able to get through to the practice by telephone

Always

I am able to get an appointment when I want one

Usually

What I liked

Receptionists
Opening hours
Location

What could be improved

I have seen my GP probably 4 times in the last few years.I must say they rush too much, appointments are only about 2 minutes long, they don't listen what I say. When I try to explain the problem they stop me saying "i know", "enough". They don't discuss anything with me, I can only sit in silence and wait to get my prescription (they don't say much, they won't ask me anything apart from "do you still smoke?"). Nothing like conversation. And when I went there last time, after they used the ostoscope to look into my ear, they had only wiped it with the paper tissue and put it back on the desk, on some documents (God bless the next patient with same problem as i had a really bad pus in my ear..)

Anything else to add?

When I visit the surgery with my son (2yr) I need to leave the buggy downstairs and walk up the stairs carrying my boy. I don't like to leave the puschair unattended but there is no lift.

See all comments about this GP that mention:

Appointments, Noise levels, Opening hours, Prescriptions

Added 17 April 12

Report this content as offensive or unsuitable

---

[1] This piece of feedback was left for a GP surgery on the NHS Choices website. This was randomly selected as a negative type of patient feedback from the ones scrolling on the front page of NHS Choices.

Vignette 2

Study A

An example[2] of feedback left on NHS Choices

Would definitely recommend this surgery

Anonymous visited [redacted] in February 2012.

Overall rating

I would recommend to a friend

I am able to get through to the practice by telephone

Always

I am able to get an appointment when I want one

Usually

I am treated with dignity and respect by the staff

Always

This GP practice involves me in decisions about my care and treatment

Always

This GP practice provides accurate and up to date information on services and opening hours

Yes

What I liked

I constantly receive excellent care from my doctor here, and the people on reception are friendly and helpful even when they're obviously busy. If I want to be seen urgently I'm happy to see any of the doctors, and so it's never a problem. All my details are on their computer anyhow so they always know what my problems are. I've been a patient here for nearly twenty years and wouldn't hesitate to recommend it. My doctor is just lovely, listens and spends time with me and I never feel a nuisance. I'm very happy as a patient at this surgery.

See all comments about this GP that mention:

Attentive, Doctors, Patient care, Reception, Surgery

Added 01 February 12

Report this content as offensive or unsuitable

---

[2] This piece of feedback was left for a GP surgery on the NHS Choices website. This was randomly selected as a positive type of patient feedback from the ones scrolling on the front page of NHS Choices.

Vignette 3

Study A

An example[3] of feedback left on iwantgreatcare.org

Report this review

Added by a patient 3 months ago (27 January, 2012)

Listening	90%
Recommend	90%
Trust	90%

Their thoughts on this service

I first saw Dr Mayet a few months after I was diagnosed with type 2 diabetes. At the time I had [redacted] diagnosed tablets and was taking them, but before seeing Dr Mayet I felt I had not been given enough information about my condition. On explaining this to Dr Mayet he directed me to various websites and ensured that I w[redacted] a course run by the local health authority. This did help as it answered all of my questions a[redacted] group were able to share their experiences. Each time I have visited Dr Mayet, I feel he has listened to any concerns I have and has done his best to ensure I am on the medication that suits me.

My only slight grumble[redacted]t I have sometimes had to wait up to 40 mins after the time of my appointment to see Dr Mayet, but this may be more due to the amount of time allowed per patient than due to Dr Mayet's time management who is obviously aware of the over-ru[redacted]apologises if it happens. This does seem to have improved lately and on my last visit i[redacted]ary 2012 I was seen 5 mins after my appointment time.

---

[3] This piece of feedback was left for a GP on the iwantgreacare.org website. This was randomly selected as patient feedback that is both negative and positive.

WMG Innovative Solutions · THE UNIVERSITY OF WARWICK · EPSRC Pioneering research and skills
